# Where antibiotic resistance mutations meet quorum-sensing

**DOI:** 10.15698/mic2014.07.158

**Published:** 2014-06-25

**Authors:** Rok Krašovec, Roman V. Belavkin, John A. Aston, Alastair Channon, Elizabeth Aston, Bharat M. Rash, Manikandan Kadirvel, Sarah Forbes, Christopher G. Knight

**Affiliations:** 1 Faculty of Life Sciences, University of Manchester, M13 9PT, UK.; 2 School of Science and Technology, Middlesex University, London NW4 4BT, UK.; 3 Statistical Laboratory, DPMMS, University of Cambridge, CB3 0WB, UK.; 4 Research Institute for the Environment, Physical Sciences and Applied Mathematics, Keele University, ST5 5BG, UK.; 5 Wolfson Molecular Imaging Centre, University of Manchester, M20 3LJ, UK.; 6 Manchester Pharmacy School, University of Manchester, M13 9PT, UK.

**Keywords:** evolution, mutagenesis, fluctuation test, autoinducer 2, autoinducer 3, stress-induced mutagenesis, DNA methylation, optimal control

## Abstract

We do not need to rehearse the grim story of the global rise of antibiotic resistant microbes. But what if it were possible to control the rate with which antibiotic resistance evolves by *de novo* mutation? It seems that some bacteria may already do exactly that: they modify the rate at which they mutate to antibiotic resistance dependent on their biological environment. In our recent study [Krašovec, *et al.* Nat. Commun. (2014), 5, 3742] we find that this modification depends on the density of the bacterial population and cell-cell interactions (rather than, for instance, the level of stress). Specifically, the wild-type strains of *Escherichia coli *we used will, in minimal glucose media, modify their rate of mutation to rifampicin resistance according to the density of wild-type cells. Intriguingly, the higher the density, the lower the mutation rate (Figure 1). Why this novel density-dependent ‘mutation rate plasticity’ (DD-MRP) occurs is a question at several levels. Answers are currently fragmentary, but involve the quorum-sensing gene *luxS* and its role in the activated methyl cycle.

The first level of questions about DD-MRP concern how the density dependence occurs. This ‘upstream’ mechanism requires the well-known quorum-sensing gene *luxS*. When *luxS* is deleted, the density dependence of their mutation rate goes away. And when wild-type cells are co-cultured with Δ*luxS* cells, their mutation rate goes up as though the Δ*luxS* cells were not contributing to the perceived cell density. This immediately suggests a role for the quorum-sensing signal, autoinducer 2 (AI-2), uniquely produced by the LuxS protein. However, the story is not that simple - adding AI-2 does not decrease the wild-type mutation rate as would be predicted if it were the mediator. Rather, it seems that the metabolic role of the LuxS protein in the activated methyl cycle is required. Indirectly replenishing this cycle by adding the amino acid aspartate to the medium functionally complements the *luxS* deletion, bringing back the DD-MRP. This still leaves open the question of what the upstream signal, by which density is perceived, actually is. In principle it could be anything acting non-cell-autonomously downstream of *luxS* via the activated methyl cycle. A prime candidate might be the elusive molecule autoinducer 3, an important regulator of virulence gene expression in enteropathogenic *E. coli*. However, while the precise identity of that molecule remains unknown, its relevance to DD-MRP is likely to remain speculative.

The second level of questions is what kind of ‘downstream’ mechanism is involved. The number of mutations in any organism results from a balance between mutation generation and DNA repair. The mechanism of this DD-MRP could therefore, in principle, involve modifying mutagenesis, DNA repair or both. The initial evidence (transcriptional analyses) does not support a role for error-prone DNA-polymerases IV and V, both known to be involved in the only other well characterised example of MRP, stress-induced mutagenesis. Among DNA repair mechanisms, indirect evidence from our analysis of published transcriptional data suggests a possible role for the *mutS* gene. Perhaps most intriguing, and also consistent with the indirect evidence, is the possibility that DNA methylation is involved in DD-MRP. This idea comes from the fact that methylation sites used by the Dam and Dcm methylases are known to be mutational hotspots. Furthermore, two key sites known to confer rifampicin resistance in *E. coli*, and hence major contributors to our mutation rate estimates, are such methylation sites (the adenine residues in the common palindrome GATC). These facts may be relevant to our observation that DD-MRP depends on *luxS*’s role in the cycle supplying methyl groups to DNA methylases. One can therefore hypothesise that differential methylation could be causally involved in DD-MRP.

The third and final ‘why’ is less mechanistic: why has DD-MRP evolved and been maintained in wild-type bacteria? The default answer has to be that DD-MRP is a by-product of something else that more directly affects the fitness of the cell. Perhaps it is a side-effect of quorum-sensing activation (whose evolutionary role is much debated in its own right). If this connection is not especially costly, and/or is genetically difficult to remove without a cost, DD-MRP could be ‘just’ an interesting quirk. This is not a particularly satisfying hypothesis: one might expect anything other than minimised mutation rates to impose a cost in terms of mutant offspring and so be selected against unless they are somehow beneficial. Also, removal of *luxS* straightforwardly removed DD-MRP without a cost we could measure in the laboratory. There are however other possibilities. We were looking for plastic mutation rates precisely because we and others are developing mathematical theory, demonstrating that MRP can be beneficial. In particular, it can be beneficial if mutation rates are minimized when an organism is as fit as it can be (i.e. at an adaptive peak), and increased when the organism is doing badly. This makes intuitive sense: when a genotype is displaced from an adaptive peak, the deleterious effects of a raised mutation rate could be outweighed by the potential for increased fitness via mutation. We have found this to occur in mathematical models of adaptive landscapes and in *in silico* evolution of DNA sequences binding particular transcription factors. Whether such beneficial MRP exists in biology, however, remains to be seen. DD-MRP requiring *luxS* does indeed give a mutation rate that is often lowest when the individuals have high fitness and increases at lower fitness. Nonetheless, the evidence is not yet there to say that this process actually does benefit the organisms, let alone that it evolved and/or is maintained because of this benefit. These are issues that need to be resolved experimentally, both over the time-scales of experimental evolution and the longer stretches of evolution among different prokaryotic, and indeed eukaryotic, microbes.

All the scientific issues above seem tractable. However, they prompt broader questions: ‘Why is DD-MRP interesting?’ and ‘Why has no-one discovered it before?’. The fluctuation test, on which our findings are based, was first used in the 1940s and rifampicin was produced in 1959. Since then, fluctuation tests using rifampicin have become commonplace for estimating mutation rates. Perhaps the previous non-discovery of DD-MRP comes down to statistics - fluctuation tests are noisy and laborious assays. Only differences in mutation rate between wild-type and mutator strains, which typically have at least an order of magnitude higher mutation rate (e.g., due to deficiency in DNA repair), can be reliably separated within a single experiment. We, by contrast, are observing variation in mutation rates of around 3 to 5-fold (see Figure 1). It seems entirely plausible that, to a bacterium, even a small increase in the chance of gaining an antibiotic resistance mutation is, biologically, highly significant. However, to detect this difference using fluctuation tests required large numbers of assays and some careful statistical models. Even then we were only really convinced of DD-MRP when it became clear that we could remove it by deleting *luxS* and reinstate it by functionally complementing that mutation. Which brings us to the question of DD-MRP’s significance - why should one care about a phenomenon close to the limits of detectability, and subtle, relative to the large fixed differences in mutation rate between wild-type and mutator strains?

**Figure 1:**
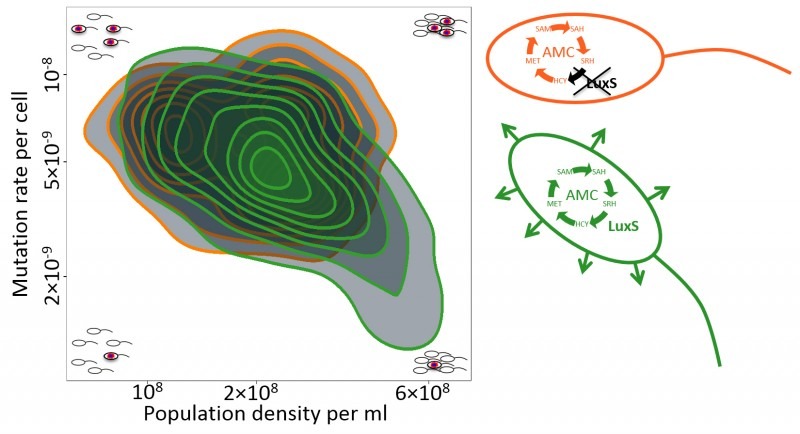
FIGURE 1: Density-dependent mutation rate plasticity in *E. coli*. The green contours indicate mutation rates for *E. coli* with a functional Activated Methyl Cycle (AMC; green cell) across a range of population densities. In the example cells in the plot corners, a blue and red explosion indicates a mutational event resulting in a rifampicin resistant cell. The orange contours correspond to *luxS *deletant cells which don’t produce the signal (arrows coming from the green cell) and have an incomplete AMC (orange cell). Contours are density plots across all the data from Krašovec *et al.* 2014 (344 data points, 77 of which correspond to the orange contours), including both *E. coli* B and K12 strains and accounting for all the effects noted there (e.g., data from the complemented Δ*luxS* mutant are included in the green area, not the orange). Abbreviations: HCY, homocysteine; MET, methionine; SAM, S-adenosylmethionine; SAH, S-adenosylhomocysteine; SRH, S-ribosylhomocysteine.

Firstly, we believe DD-MRP is interesting because it connects previously disparate areas of microbiology: mutagenesis and quorum-sensing. A vast amount of knowledge has been built up in both areas and DD-MRP opens a bridge between the two. It’s interesting too as an evolutionary mechanism, particularly where that relates to mathematical models and theory. The independence of mutational effects from the environment in which they arise has been a hard-won insight. Nonetheless, evolution depends ultimately upon mutations and intimately upon the environment. Links between the two therefore have the potential to affect the realized trajectory of evolution. Finally, there is the interest of what DD-MRP might mean practically. If mutation rates in general, and those to antibiotic resistance in particular, can be manipulated via a quorum-sensing-like mechanism, it opens up fascinating possibilities for how microbes might be manipulating each other and ultimately, how humans might manipulate them too. In particular, there are many circumstances outside the laboratory when *E. coli* cells find themselves at low densities, for instance, when treated by antibiotics in the human gut or when first infecting a host. If it were possible to develop a molecule that prevents *E. coli* cells from recognizing their scarcity, our results suggest that this could reduce the rate at which antibiotic resistance arises. If administered with existing antibiotics, such a molecule might increase or prolong their efficacy. Such a mutation rate minimization approach could perhaps result in new tools to tackle the rise in antibiotic resistance. After all, attine ants have apparently succeeded in suppressing microbial pathogens using bacterial antibiotics for millennia without obviously ‘inventing’ new classes of antibiotic. Perhaps the identification of density-dependent mutation rates will play a part in humans learning the same trick.

